# Antimicrobial, Cytotoxic, and Anti-Inflammatory Activities of *Tigridia vanhouttei* Extracts

**DOI:** 10.3390/plants12173136

**Published:** 2023-08-31

**Authors:** Jorge L. Mejía-Méndez, Ana C. Lorenzo-Leal, Horacio Bach, Edgar R. López-Mena, Diego E. Navarro-López, Luis R. Hernández, Zaida N. Juárez, Eugenio Sánchez-Arreola

**Affiliations:** 1 Laboratory of Phytochemistry Research, Chemical Biological Sciences Department, Universidad de las Américas Puebla, Ex Hacienda Sta. Catarina Mártir S/N, San Andrés Cholula 72810, Mexico; luisr.hernandez@udlap.mx; 2 Division of Infectious Diseases, Faculty of Medicine, University of British Columbia, Vancouver, BC V6H 3Z6, Canada; anacecylole@gmail.com; 3 Tecnologico de Monterrey, Escuela de Ingeniería y Ciencias, Campus Guadalajara, Av. Gral. Ramón Corona No 2514, Colonia Nuevo México, Zapopan 45121, Mexico; edgarl@tec.mx (E.R.L.-M.); diegonl@tec.mx (D.E.N.-L.); 4 Chemistry Area, Deanship of Biological Sciences, Universidad Popular Autónoma del Estado de Puebla, 21 Sur 1103 Barrio Santiago, Puebla 72410, Mexico; zaidanelly.juarez@upaep.mx

**Keywords:** traditional medicine, Iridaceae, *Iris*, *Tigridia vanhouttei*, biological activities

## Abstract

In this work, bulb extracts of *Tigridia vanhouttei* were obtained by maceration with solvents of increasing polarity. The extracts were evaluated against a panel of pathogenic bacterial and fungal strains using the minimal inhibitory concentration (MIC) assay. The cytotoxicity of the extracts was tested against two cell lines (THP-1 and A549) using the MTT assay. The anti-inflammatory activity of the extracts was evaluated in THP-1 cells by measuring the secretion of pro-inflammatory (IL-6 and TNF-α) and anti-inflammatory (IL-10) cytokines by ELISA. The chemical composition of the extracts was recorded by FTIR spectroscopy, and their chemical profiles were evaluated using GC-MS. The results revealed that only hexane extract inhibited the growth of the clinical isolate of *Pseudomonas aeruginosa* at 200 μg/mL. Against THP-1 cells, hexane and chloroform extracts were moderately cytotoxic, as they exhibited LC_50_ values of 90.16, and 46.42 μg/mL, respectively. Treatment with methanol extract was weakly cytotoxic at LC_50_ 443.12 μg/mL against the same cell line. Against the A549 cell line, hexane, chloroform, and methanol extracts were weakly cytotoxic because of their LC_50_ values: 294.77, 1472.37, and 843.12 μg/mL. The FTIR analysis suggested the presence of natural products were confirmed by carboxylic acids, ketones, hydroxyl groups, or esters. The GC-MS profile of extracts revealed the presence of phytosterols, tetracyclic triterpenes, multiple fatty acids, and sugars. This report confirms the antimicrobial, cytotoxic, and anti-inflammatory activities of *T. vanhouttei*.

## 1. Introduction

Currently, human health is being threatened by infections caused by drug-resistant microorganisms, such as bacteria and fungi, and high-incident types of cancer [1,2].

According to the Centers for Disease Control and Prevention (CDC), it has been documented that resistance to current antimicrobials has resulted in more than 2.8 million infections and 35,000 deaths in the United States of America (USA) [3]. Multi-drug-resistant bacteria related to these events include members of the ESKAPE (*Enterococcus faecium*, *Staphylococcus aureus*, *Klebsiella pneumoniae*, *Acinetobacter baumannii*, *Pseudomonas aeruginosa*, and *Enterobacter* species) pathogens.

In comparison to other bacteria, ESKAPE pathogens are the main cause of nosocomial infections since they can evade the activity of antibacterial agents due to the development of several drug-resistance mechanisms, for example, drug-binding site alteration, changes in the permeability of drugs, and aberrations in drug efflux transporters [4]. Similar effects are observed for drug-resistant fungi strains that belong to the *Aspergillus*, *Candida*, *Cryptococcus*, and *Pneumocystis* genera [5], which can lead to invasive fungal infections in the bloodstream, lungs, brain, and skin [6].

Cancer arises from the uncontrolled proliferation and growth of cells, and it is classified in view of its tissue or cell of origin. Lung cancer (LC) can originate from the central area and peripheric regions of the lungs [7]. In contrast to other types of cancer, LC is characterized by its invasiveness, aggressiveness, and high prevalence worldwide [8]. Epidemiologically, LC is the most common type of cancer diagnosed, and the leading cause of cancer mortality among men and women [9]. Another highly prevalent type of cancer is acute myeloid leukemia (AML). AML constitutes a heterogeneous disorder that arises from the clonal expansion of myeloid progenitors in peripheral blood and bone marrow. This results in bone marrow failure and hampers erythropoiesis [10]. Among leukemias, it is estimated that AML accounts for 80% of all cases in the adult population and more than 10,000 deaths over the last years [11]. The latter represented 1.8% of all cancer deaths in the United States [12].

Despite their molecular and cellular differences, LC and AML are treated with chemotherapy, radiotherapy, immune therapy, and targeted therapy regimens. Despite their possible efficacy, their use is prone to failure due to their limited specificity, poor solubility, the possibility of relapse, and numerous toxicities that can affect the cardiovascular, pulmonary, and musculoskeletal systems [13]. Since the registered numbers for infections caused by pathogenic bacteria and fungi, and patients diagnosed with LC or AML are expected to increase in the next decades, it is imperative to continue exploring, considering, and evaluating sources with potential biological activity.

Traditional medicine combines knowledge, practices, and experiences from indigenous cultures. In this discipline, compounds isolated from animals, microorganisms, or plants are used to treat, prevent, maintain, or improve human health [14]. Nowadays, natural products isolated from sources used in traditional medicine are broadly investigated through integrative and interdisciplinary approaches to continue developing relevant information regarding their use in the pharmaceutical, food, and healthcare industries. This has been reviewed in an innovative platform known as the International Natural Product Sciences Taskforce (INPST), which considers medicinal plants as important sources of bioactive molecules for modern medicine [15]. Medicinal plants are representative sources of secondary metabolites that can exert multiple therapeutic properties such as antimicrobial, antioxidant, antidiabetic, anti-inflammatory, etc. The family Iridaceae includes many flowering plants distributed in South Africa, the Eastern Mediterranean, and Central America [16].

In the past, species that belong to this family have been used to prepare decoctions, pastes, syrups, and extracts to treat muscle pains, respiratory syndromes, neurological disorders, gastrointestinal diseases, and cancers [17]. The genus *Iris* is the largest genus of the Iridaceae family, and its species are differentiated because of their violet-like scent and broad presence in North America, Europe, and Asia [18]. In traditional medicine, species from the genus *Iris* have been considered to treat infections caused by bacteria, viruses, cancer, and inflammatory disorders [18]. The documented biological activities are attributed to the bioactive natural products that they contain such as spiroiridals, flavonoids, triterpenoids, and xanthones [16].

Taxonomically, the genus *Iris* is subdivided into the following four subfamilies: Ixoideae, Isophysidoideae, Nivenioideae, and Iridoideae [19]. The latter is divided into the following tribes: Mariceae, Irideae, Sisyrinchieae, and Tigridieae [19]. The tribe Tigridieae constitutes a monophyletic group that is organized into thirteen genera, sixty-six species, and seven subspecies and is widely distributed in North America [20]. Over the last decades, the phytochemical composition of some *Tigridieae* species has been reported. For example, iridals such as spiroiridal, belamcandal, and 16-hydroxyiridal have been identified in the essential oil of *T. pavonia* [21]. Moreover, glucosyl xanthones, such as mangiferin, have been identified in methanol extracts from *T. alpestris* [22]. However, to our knowledge, for other *Tigridieae* species, such as *T. vanhouttei*, no biological activities or chemical composition have been reported.

Continuing with our research program about scientifically validating the medicinal use of plants, this study aimed to investigate the antimicrobial activity of extracts from *T. vanhouttei* against a panel of human bacterial and fungal pathogens. The cytotoxicity of extracts was tested against human-derived macrophage THP-1 cells and A549 cells. To evaluate the chemical composition of extracts, FTIR spectroscopy was used, whereas GC-MS analyses were considered to assess their chemical profile.

## 2. Results and Discussion

### 2.1. FTIR Analysis

**Figure 1:**
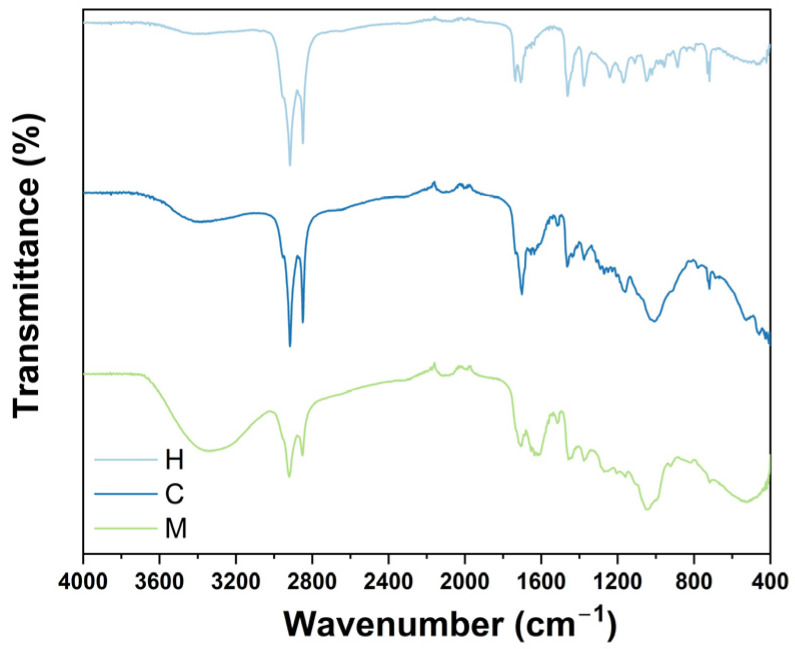
FTIR analysis of hexane (H), chloroform (C), and methanol (M) extract from *T. vanhouttei*.

In contrast to other spectroscopy techniques, FTIR spectroscopy is based on the absorption of infrared light by proteins, lipids, carbohydrates, and fatty acids. For plant extracts, FTIR analyses are utilized to preliminary characterize their chemical composition by determining the presence of multiple functional groups (e.g., ketones, esters, and carbonyl groups) that can be related to their phytoconstituents. As presented in Figure 1, hexane, chloroform, and methanol extracts exhibit similar bands within the 4000 to 400 cm^−1^ range. Initially, it can be noted that the three extracts present two sharp bands at 2920 and 2835 cm^−1^, which are related to the symmetrical and asymmetrical stretching of C-H bonds. As expected, the FTIR spectrum of methanol extract displays a broad band at 3300 cm^−1^, which corresponds to the stretching of the O-H bond possibly from the methanol used to prepare this extract or phenolic compounds. Moreover, the three extracts share a series of peaks located from 1700 to 900 cm^−1^ that can be related to the presence of carboxylic acids, aromatic amines, and alkenes. To record the chemical profile of extracts from *T. vanhouttei*, GC/MS was used.

### 2.2. GC-MS Analysis

Among chromatography techniques, GC-MS represents a robust and sensitive approach for analyzing chemical and biological samples. In the study of plant extracts, using GC-MS predominantly enables the determination of volatile compounds with low molecular weight [23]. Using this technique, we assessed the phytochemical content of extracts from *T. vanhouttei*; see Table 1. Chromatograms are presented in the Supplementary Material (Figures S1–S3).

### 2.3. Antimicrobial Activity

Plant extracts constitute an attractive alternative to evaluate and obtain innovative antimicrobial agents. The advantages of plant extracts over current antimicrobials rely on their intrinsic biological activity, synergistic effects, limited toxicity, and capacity to suppress drug-resistance mechanisms [24]. However, their activity can be limited due to possible antagonism between their phytoconstituents [25].

As indicated in Table 2, results revealed that only hexane extract inhibited growth of the clinical isolate of *P. aeruginosa* at 200 μg/mL (Table 2). No antibacterial activity was observed during treatment with chloroform and methanol extract. In addition, extracts did not exert antifungal activity against the tested strains at the proposed concentrations (50,100, 150, and 200 μg/mL).

In the healthcare system, *P. aeruginosa* is considered an opportunistic pathogen that can infect patients with burn wounds, immunodeficiency, or cystic fibrosis [26]. In contrast to other gram-negative strains, *P. aeruginosa* has represented a serious source of mortality and morbidity among long-term care hospitals and intensive care units over the last few years [27].

The activity of hexane extract against this strain can be attributed to the presence of palmitic and oleic acids, which can display antibacterial activity due to their ability to inhibit the activity of essential components for bacterial fatty acid biosynthesis, such as the enoyl-acyl carrier protein reductase component [28]. In addition, phytosterols such as stigmasterol have been reported to exhibit bactericidal activity against gram-negative bacteria due to their ability to modify bacterial membrane composition [29]. These findings suggest the potential use of *T. vanhouttei* extracts to treat infections caused by clinical isolates of *P. aeruginosa*.

### 2.4. Cytotoxic Activity

Cytotoxicity assays are required to determine the potential toxicity of bioactive substances before their consideration in the development of pharmaceutical formulations [30]. This is often applied to plant extracts or isolated natural products against several models of cancer cell lines.

Here, we tested the cytotoxicity of extracts from *T. vanhouttei* against the THP-1 and the A549 cell lines. The former is a human leukemia monocytic cell line that is widely cultured to investigate the molecular and cellular functionality of monocytes or macrophages [31] and screen the toxicity of candidate molecules against them [32]. The latter comprehends human alveolar basal epithelial cells that are broadly used to assess the functionality of alveolar cells [33], and the potential use of plant extracts or synthetic molecules against lung cancer [34,35].

**Figure 2:**
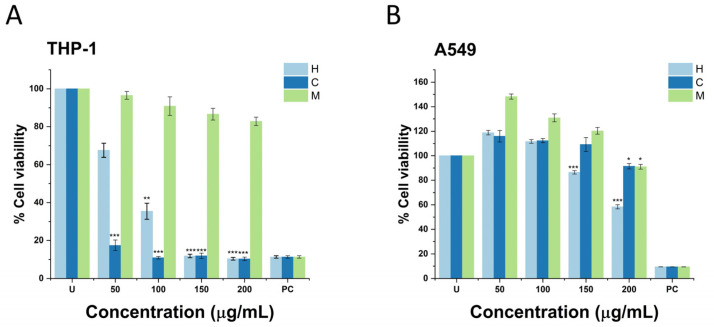
% of cell viability of human-derived macrophage (**A**) THP-1 and (**B**) A549 cells against treatment with 50, 100, 150, and 200 μg/mL of *T. vanhouttei* extracts. U, untreated cells; H, hexane extract; C, chloroform extract; M, methanol extract. PC represents SDS, which was used as a positive control. Shown is the mean ± S.D. of three independent experiments. * *p* values < 0.05.

As represented in Figure 2A, treatment with hexane and chloroform extracts decreased the viability of THP-1 cells in a dose-dependent manner (50, 100, 150, and 200 μg/mL). Initially, it can be observed that treatment with 50 μg/mL of hexane extract resulted in 22.07% of THP-1 cell death, whereas treatment with 100 μg/mL resulted in 71.22% cell death (*p* < 0.005). Against treatment with 150 and 200 μg/mL, 88.14 and 89.61% of cell death were registered (*p* < 0.0005), respectively. This phenomenon was more evident with chloroform extract treatment.

In Figure 2A, it can be noted that treatment with 50 μg/mL of chloroform extract resulted in 82.56% cell death (*p* < 0.005). During treatment with 100, 150, and 200 μg/mL of chloroform extract, 88.06, 89.09, and 89.69% of THP-1 cell deaths were recorded, respectively (*p* < 0.0005). In the same figure, it can be observed that the cytotoxicity of methanol extract against the THP-1 cell line was weak since the cells continued to proliferate at 50 μg/mL. However, at 100, 150, and 200 μg/mL, THP-1 cells presented a 9.15, 20.39, and 27.01% in cell death. Despite the importance of these results, the cytotoxicity of plant extracts at different concentrations against other cell lines can vary, which in this case was observed against the A549 cell line.

It can be noted in Figure 2B, that treatment with 50 or 100 μg/mL of hexane extract was not cytotoxic against A549 cells. In fact, cells continued to proliferate after 24 h of exposure to treatment. For the same extract, treatment with 150 and 200 μg/mL resulted significantly in the death of 13.58 and 41.61% A549 cells, respectively (*p* < 0.005). Even though treatment with chloroform extract at 50, 100, and 150 μg/mL did not induce the death of A549 cells, treatment with 200 μg/mL occurred in 8.55% of A549 cell deaths. At the same concentration, treatment with 200 μg/mL of methanol extract resulted in 8.96% cell death. Only statistical differences were registered for treatment with hexane extract against the A549 cell line.

In view of the results obtained in both cytotoxic assays, we assessed the LC_50_ for extracts against each cell line. Against THP-1 cells, the calculated LC_50_ values of the hexane, chloroform, and methanol extract were 90.16, 46.42, and 443.12 μg/mL, respectively. Against the A549 cell line, hexane, chloroform, and methanol extract presented the following LC_50_ values: 294.77, 1472.37, and 843.12 μg/mL. Following the National Cancer Institute (NCI) of the United States, extracts can be considered moderately cytotoxic against the THP cell line, whereas against the A549 cell line, their cytotoxicity is considered weak [36]. The LC_50_ values calculated for each cell line are compiled in Table 3.

Plant extracts can display distinct biological activities through bioactive secondary metabolites. Even though the biological activities of *Tigridia* species are unknown, recent studies about medicinal plants, such as *Polygonum hydropiper* L., have demonstrated that sterols, such as β-sitosterol, can decrease the viability of breast and cervical cancer cell lines at 1 mg/mL [37]. For the same sterol, its capacity to interfere with the apoptosis, cell cycle, cell signaling pathways, invasion, and survival of lung, stomach, colon, and leukemia cells has been reviewed recently [38].

In other studies, where the cytotoxicity of natural products, such as furanocoumarins, has been investigated, it was reported that isopsoralen could exert cytotoxic activity against human hepatoma cells at the micromolar range (10–200 mM) in a time-dependent manner [39]. Regarding the cytotoxicity of fatty acids, it has been unveiled that palmitic acid can enhance the generation of reactive oxygen species (ROS), induce apoptosis by promoting the activity of caspase 3, decrease mitochondrial membrane potential, and cause cell damage among hepatocyte cell cultures in the millimolar range (0.125–2 mmol/L). In the same study, the activity of oleic acid was tested; however, no significant changes were observed [40]. The presence of these compounds among extracts from *T. vanhouttei* can explain the observed cytotoxicity.

### 2.5. Anti-Inflammatory Activity

Inflammation constitutes a complex biological response induced by pathogens, toxic compounds, or damaged cells [41]. Depending on its progression, inflammation can lead to tissue damage or disease development. Common inflammation-related diseases include atherosclerosis, rheumatoid arthritis, diabetes, and cancer [42]. It is well-known that plant extracts constitute sources of promising bioactive compounds that can interfere with inflammatory processes. Therefore, there is a need to continue exploring them.

**Figure 3:**
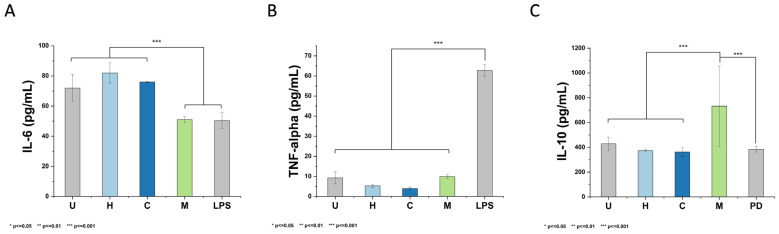
Immunological response of *T. vanhouttei* extracts: hexane (H), chloroform (C), and methanol (M) on human-derived THP-1 cells using ELISA for (**A**) IL-6, (**B**) TNF-α, and (**C**) IL-10. Untreated cells (U); PD, prednisone (positive control for anti-inflammatory analysis); LPS, lipopolysaccharide (positive control for inflammatory analysis). Shown is the mean ± S.D. of three independent experiments.

The capacity of extracts from *T. vanhouttei* to elicit an inflammatory response in THP-1-derived macrophages is presented in Figure 3. Results revealed that THP-1 cells treated with LPS or methanol extract decreased IL-6 levels to 50.33 ± 5.49 and 50.99 ± 1.98 pg/mL, respectively. Conversely, cells treated with 50 μg/mL of hexane and chloroform extracts significantly exhibited 81.94 ± 6.68 and 75.95 ± 0.41 IL-6 levels, respectively (*p* < 0.001). IL-6 is a pleiotropic cytokine that is commonly associated with inflammatory processes when dysregulated. However, it is also involved in the hematopoiesis process, acute phase responses against infections and tissue injuries, immune cell functionalities, and immune reactions [43]. The obtained results suggest the potential use of hexane and chloroform extracts from *T. vanhouttei* as anti-inflammatory agents that modulate the secretion of IL-6 to treat bone destruction disorders [44] or regulate metabolic and cardiovascular events [45]. The observed phenomenon can be different against other pro-inflammatory cytokines, such as TNF-α.

TNF-α is also a pleiotropic cytokine that can regulate inflammatory responses but is commonly associated with the progression of both autoimmune and inflammatory diseases [46]. According to Figure 3B, treatment with LPS significantly promoted the secretion of TNF-α (62.71 ± 2.88 pg/mL), whereas treatment with 50 μg/mL of hexane, chloroform, or methanol extract exhibited the following TNF-α levels: 5.28 ± 0.70, 3.97 ± 0.68, and 9.93 ± 0.97 pg/mL, respectively. Since the levels of TNF-α were not enhanced during treatment with extracts from *T. vanhouttei*, these results suggest their anti-inflammatory activity. To continue evaluating the anti-inflammatory activity of extracts, the secretion of IL-10 was investigated.

Among anti-inflammatory cytokines, IL-10 mediates the host’s anti-inflammatory response against external stimuli, stimulates immune cells’ activation, differentiation, and proliferation, and inhibits non-specific immunological responses [47]. As depicted in Figure 3C, cells treated with 50 μg/mL of hexane extract presented no significant levels of IL-10 (373.28 ± 10.09 pg/mL) in contrast to prednisone, which was used as a positive control. Comparably, no significant differences were observed during treatment with 50 μg/mL chloroform extract, as it exhibited 362.03 ± 36.58 pg/mL IL-10 levels. This effect varied with methanol extract treatment as it showed a significant IL-10 level: 732.30 ± 324.87 pg/mL. The observed anti-inflammatory activities of extracts from *T. vanhouttei* can be due to their phytochemical content.

Plant extracts possess anti-inflammatory activities due to their ability to interfere with oxidation-reduction reactions, modulate cell signaling pathways involved in inflammatory processes, and interfere with reactive species generation [48].

In the case of hexane and chloroform extracts, their anti-inflammatory activity can be due to the presence of stigmasterol, which has been observed to exert this effect in murine models [49] and prevent the generation of pro-inflammatory cytokines [50].

In addition, the potential anti-inflammatory activity of chloroform extract can be attributed to the existence of campesterol, which has been reported to interfere with the release of TNF-α in a dose-dependent manner (25–200 mM) [51]. Compounds such as β-sitosterol and stigmasterol can also inhibit the production of TNF-α at the same concentration range [51] and promote the secretion of IL-10 [52]. The presence of β-sitosterol can be related to the capacity of methanol extract to induce the secretion of IL-10.

## 3. Materials and Methods

### 3.1. Plant Material and Extract Preparation

Bulbs from *T. vanhouttei* were collected in Texcoco, Estado de México (19°29′ N 98°53′ W). Specimens were identified by the biologist Lilián López-Chávez and deposited with voucher number 36230 at the herbarium of Universidad Autónoma de Chapingo (Carr. Federal México-Texcoco, 56230, Texcoco, Estado de Mexico). For extract preparation, 500 g of bulbs were dried at room temperature, powdered using a mechanical blender, and progressively macerated with *n*-hexane, chloroform, and methanol for 72 h at room temperature. Mixtures were manually mixed at the beginning of this process. The time of extraction was selected since it is the time recommended to efficiently extract bioactive components from plants [53]. The ratio of this process was 0.33 g of plant per milliliter of solvent. Solvents were evaporated under reduced pressure to dryness using a rotary evaporator (Heidolph Laborota 4000; Schwabach, Germany). Extracts were collected and maintained under refrigeration for further evaluation.

### 3.2. FTIR Evaluation

FTIR spectroscopy has been widely used to amplify the knowledge regarding the identification and differentiation between the chemical composition of extracts from foods, fruits, nanomaterials, and plant extracts by providing a characteristic fingerprint [54]. To evaluate the chemical composition of extracts from *T. vanhouttei,* a Cary 630 Fourier-transform infrared (FTIR) spectrometer (Agilent Technologies, Santa Clara, CA, USA) was used. An ethanol solution (100% *v*/*v*) was added to clear the detection diamond, and background spectra were determined without samples at 25 °C. To perform sample analysis, 20 mg of each extract was placed, and ethanol was used again to clean the detection diamond after each measurement. Spectra were recorded within the 4000 to 400 cm^−1^ wavenumber region. Experiments were performed in triplicate.

### 3.3. Analysis of Phytoconstituents by GC-MS

The extracts’ chemical profiles of *T. vanhouttei* were established using a Varian CP-3800 gas chromatograph coupled to a Varian 1200 quadrupole mass spectrometer. The extracts were analyzed according to published protocols [55]. Briefly, 1 µL of samples prepared at 1% (*w*/*v*) chloroform were injected into a Factor Four capillary column: VF-5MS (5% phenylmethyl polysiloxane–95% polydimethylsiloxane; Agilent Technologies), 30 m × 0.25 mm, and 0.25 µm thickness. The separation of phytoconstituents was achieved by using helium as the carrier gas (1 mL/min flow rate) at the following gradient temperature: 60 °C for 2 min, 120 °C for 16 min, 160 °C for 15 min, 180 °C for 15 min, 200 °C for 10 min, 230 °C for 15 min, 290 °C for 20 min, and 300 °C for 30 min. The extracts’ components were determined according to their fragmentation patterns and retention times by consulting the National Institute of Standards and Technology Mass Spectral (NIST-MS) database. The relative percentage of phytoconstituents was registered based on the total area of the peaks.

### 3.4. Strains and Culture Media

In this work, a panel of gram-positive and gram-negative bacteria was used. Gram-positive bacteria included *Staphylococcus aureus* (ATCC 25923) and methicillin-resistant *Staphylococcus aureus* (MRSA) (ATCC 700698) strains. Gram-negative bacteria included *Acinetobacter baumannii* (ATCC BAA-747), *Escherichia coli* (ATCC 25922), and *Pseudomonas aeruginosa* (ATCC 14210) strains. Clinical isolates of *A. baumannii* and *P. aeruginosa* were also tested. In addition, this study evaluated the *Trichophyton mentagrophytes* (ATCC 9533) strain. The pathogenic fungi *Cryptococcus neoformans* var. *grubii* (provided by Dr. Karen Bartlett, University of British Columbia, BC, Canada) and *Candida albicans* (ATCC 10231) strains were also evaluated. Bacterial strains were cultured in Mueller–Hinton broth (Becton and Dickinson (B&D)) at 37 °C in a shaker, whereas Sabouraud broth (B&D) was used for fungal strains at 28 °C.

### 3.5. Minimal Inhibitory Concentration (MIC) Assay

Following previous protocols [56], 50, 100, 150, and 200 µg/mL of *T. vanhouttei* extracts dissolved in DMSO were tested in a 96-well plate at a final volume of 100 µL/well of Mueller–Hinton or Sabouraud broth. Microbial strains were prepared to have a final optical density of 0.05 at 600 nm. MICs were defined as the concentration of the extracts at which no microbial growth was observed. For bacteria, treatment with amikacin or gentamicin was considered the positive control, whereas treatment with DMSO was used as a negative control. For fungi, amphotericin and terbinafine were used as positive controls, and DMSO remained as a negative control. All experiments were performed in triplicate.

### 3.6. Cell Culture

The cytotoxicity of *T. vanhouttei* extracts was analyzed using human-derived THP-1 monocytic (ATCC TIB-202) and pulmonary A549 (CCL-185) cells. The THP-1 cell line was cultured using an RPMI 1640 (Hyclone, GE Healthcare, Logan, UT, USA) medium supplemented with 2 mmol L-glutamine (Stem cell Technologies, Vancouver, BC, Canada) and 5% fetal bovine serum (FBS) (Hyclone) and differentiated using 20 ng/mL of phorbol 12-myristate 13-acetate (PMA, Sigma). The A549 (CCL-185) cell was cultured using Dulbecco’s Modified Eagle’s Medium (DMEM; Gibco, Carlsbad, CA, USA) containing 10% FBS and 100 µg/mL streptomycin. Both cell lines were maintained in a humidified atmosphere supplemented with 5% CO_2_ at 37 °C.

### 3.7. Cytotoxicity Assay

The cytotoxicity of *T. vanhouttei* extracts was analyzed using human-derived THP-1 monocytic (ATCC TIB-202) and pulmonary A549 (CCL-185) cells [57]. The THP-1 cell line was cultured using an RPMI 1640 (Hyclone, GE Healthcare, Logan, UT, USA) medium supplemented with 2 mmol L-glutamine (Stem cell Technologies, Vancouver, BC, Canada) and 5% fetal bovine serum (FBS) (Hyclone) and differentiated using 20 ng/mL of phorbol 12-myristate 13-acetate (PMA, Sigma). The A549 cell line was cultured using Dulbecco’s Modified Eagle’s Medium (Gibco, Carlsbad, CA, USA) containing 10% FBS, and 100 µg/mL streptomycin. To perform the cytotoxicity assay, 1 × 10^5^ of THP-1 or A549 cells were dispensed (per well) in a 96-well plate in a final volume of 100 µL, respectively. The plate was incubated at 37 °C and supplemented with an atmosphere of 5% CO_2_. The next day, the medium was changed, and the cells were treated with extracts at concentrations ranging from 50 to 200 µg/mL. The plate was incubated under the same conditions, and the next day, 25 µL of an MTT (3-(4,5-dimethylthiazol-2-yl)-2,5-diphenyltetrazolium bromide, Sigma) solution (5 mg/mL) was added per well, and the plate was incubated for 4 h at 37 °C under a supplemented atmosphere with 5% CO_2_. Formazan crystals were dissolved with 100 µL of extraction buffer, which was prepared with 20% (*w*/*v*) of sodium dodecyl sulfate (SDS) in a warm solution of dimethyl formamide at 50% containing 2.5% HCl and 2.5% acetic acid. The plate was placed in an incubator overnight at 37 °C. The next day, the absorbance was measured at 570 nm utilizing a plate reader (Epoch, BioTek). Untreated cells were considered negative controls, whereas cells treated with SDS (2%) were considered positive controls. The half-maximal lethal concentration (LC_50_) was calculated by plotting the extract concentrations against the percentage of damaged cells. The percentage of cell death for both cell lines was calculated by normalizing the absorbance of untreated cells to 100% and considering published reports where the cytotoxicity of extracts has been assessed [58].

### 3.8. Inflammatory Assay

The inflammatory assay was performed following published protocols [57]. THP-1 cells, differentiated with PMA, were dispensed at a final concentration of 1 × 10^5^ cells/well using a 96-well plate. Considering instructions from the manufacturer, the secretion of the anti-inflammatory cytokine IL-10 and the pro-inflammatory cytokine IL-6 and TNF-α was measured using commercial kits (B&D). As controls, cells treated with DMSO were used as a negative control, whereas cells treated with 1 µg/mL of lipopolysaccharide (LPS) from *Escherichia coli* (Sigma-Aldrich) were considered the positive control. For the measurement of the secretion of IL-10, prednisone (PD) was used as a positive control. Untreated cells were used as a negative control. Readings were determined utilizing a microplate reader at 450 nm. Experiments were performed in triplicate.

### 3.9. Statistical Analysis

To assess significant statistical differences between data obtained from cell viability, a two-way analysis of variance (ANOVA) followed by Tukey’s mean separation test was applied using OriginPro 2023 data processing software (OriginLab, Northampton, MA, USA).

## 4. Conclusions

This work reported, for the first time, the antimicrobial, cytotoxic, and anti-inflammatory activities of extracts from *T. vanhouttei*. Among the tested extracts, only hexane extract inhibited the growth of the clinical isolate of *P. aeruginosa*. The cytotoxicity of the obtained extracts was tested against representative cancer cell lines from leukemia and lung cancer. Even though the obtained extracts exhibited moderate cytotoxic activity against THP-1 cells and weak cytotoxic activity against A549 cells, these results suggest their potential use against cancer. Regarding their anti-inflammatory activity, methanol extract promoted the secretion of IL-10, whereas hexane and chloroform extract did not elicit the secretion of TNF-α, suggesting their potential anti-inflammatory effect. The recorded biological activities of extracts can be attributed to the various fatty acids and sterols that they contain, which were preliminary identified by FTIR spectroscopy and confirmed by GC/MS analysis. Taking together these results, the novelty of this research relies on demonstrating, for the first time, the therapeutic application of Tigridieae species using in vitro models. The findings of this work can have several applications in pharmacognosy and pharmacotherapy.

## Figures and Tables

**Table 1:** GC/MS analysis of extracts from *T. vanhouttei*.

Extract	Rt (min)	R Match	Match	%	Compound
Hexane	42.493	851	869	3.62	Palmitic acid
	46.386	864	811	0.04	Myristic acid
	46.717	791	783	0.54	Ascorbic acid
	54.295	939	895	13.29	Linolelaidic acid
	54.664	920	908	12.27	Oleic acid
	101.676	875	814	1.34	Octacosane
	115.255	873	844	2.58	Nonacosane
	120.443	779	757	0.77	Isopsoralen
	122.133	730	721	0.53	β-Stigmasterol
	126.025	813	799	1.17	ε-Sitosterol
	127.875	866	811	2.22	Untriacontane
	133.475	773	703	0.42	Sitostenone
Chloroform	67.704	878	869	5.26	Hexadecanoic acid
	74.340	862	809	8.19	Tetradecanoic acid
	88.372	872	820	5.01	Linolelaidic acid
	90.104	831	800	1.73	Oleic acid
	95.842	790	764	6.11	Stearic acid
	129.154	771	739	2.16	Arachidic acid
	168.336	829	794	1.21	Methyl lignocerate
	180.281	730	707	1.11	Campesterol
	181.254	747	742	1.58	Stigmasterol
Methanol	43.475	927	843	27.51	Tridecanoic acid methyl ester
	46.164	827	794	33.26	Pentadecanoic acid
	55.765	871	854	9.93	Linoleic acidmethyl ester
	56.264	805	787	2.86	Myristoleic acid
	57.959	877	801	2.18	Methyl tetradecanoate
	58.412	853	801	7.35	Stearolic acid
	58.728	749	703	5.03	Oleic acid
	100.201	782	756	1.93	β-Sitosterol

**Table 2:** Antimicrobial activity of extracts from *T. vanhouttei* expressed as the minimal inhibitory concentration (μg/mL).

Extract	Bacteria								Fungi		
	AB	EC	MRSA	PA	LM	SA	AB_c_	PA_c_	CA	CN	TM
Hexane	R	R	R	R	R	R	R	200	R	R	R
Chloroform	R	R	R	R	R	R	R	R	R	R	R
Methanol	R	R	R	R	R	R	R	R	R	R	R

Abbreviations: AB, *Acinetobacter baumannii*; EC, *Escherichia coli*; MRSA, methicillin-resistant *Staphylococcus aureus*; PA, *Pseudomonas aeruginosa*; LM, *Listeria monocytogenes*; SA, *Staphylococcus aureus*; AB_c_, *Acinetobacter baumannii* clinical isolate; PA_c_, *Pseudomonas aeruginosa* clinical isolate; CA, *Candida albicans*; CN, *Cryptococcus neoformans*; TM, *Trichophyton mentagrophytes*; R, resistant.

**Table 3:** LC_50_ values of hexane, chloroform, and methanol extract from *T. vanhouttei* against THP-1 and A549 cell lines. Concentrations are expressed in μg/mL.

Extract	THP-1	A549
Hexane	90.16	294.77
Chloroform	46.42	1472.37
Methanol	443.12	843.12

## Data Availability

Experimental data generated in this work can be requested from the authors for correspondence.

## Supplementary Materials

The following supporting information can be downloaded at https://www.mdpi.com/article/10.3390/plants12173136/s1, Figure S1: Chromatogram of hexane extract from *T. vanhouttei*. Figure S2: Chromatogram of chloroform extract from *T. vanhouttei*. Figure S3: Chromatogram of methanol extract from *T. vanhouttei*.
