# Supplementary material for: Antimicrobial, Cytotoxic, and Anti-Inflammatory Activities of Tigridia vanhouttei Extracts
Source: Plants (Basel). 2023 Aug 31;12(17):3136. doi: 10.3390/plants12173136 (PMC10489859; doi:10.3390/plants12173136)

Antimicrobial, Cytotoxic, and Anti-inflammatory Activities of *Tigridia vanhouttei* Extracts

Jorge L. Mejía-Méndez[1,†,*], Ana C. Lorenzo-Leal[2,†], Horacio Bach[2,†,*], Edgar R. López-Mena[3], Diego E. Navarro-López[3], Luis R. Hernández[1], Zaida N. Juárez[4], Eugenio Sánchez-Arreola[1,*]

[1] Laboratory of Phytochemistry Research, Chemical Biological Sciences Department, Universidad de las Américas Puebla, Ex Hacienda Sta. Catarina Mártir S/N, San Andrés Cholula 72810, Mexico; luisr.hernandez@udlap.mx (L.R.H.).

[2] Division of Infectious Diseases, Faculty of Medicine, University of British Columbia, Vancouver, BC V6H 3Z6, Canada; anacecylole@gmail.com (A.C.L.-L.).

[3] Tecnologico de Monterrey, Escuela de Ingeniería y Ciencias, Campus Guadalajara, Av. Gral. Ramón Corona No 2514, Colonia Nuevo México, Zapopan 45121, Jalisco, Mexico; edgarl@tec.mx (E.R.L.-M.); diegonl@tec.mx (D.E.N.-L.).

[4] Chemistry Area, Deanship of Biological Sciences, Universidad Popular Autónoma del Estado de Puebla, 21 Sur 1103 Barrio Santiago, Puebla 72410, Mexico; zaidanelly.juarez@upaep.mx (Z.N.J.).

[†] These authors contributed equally to this work.

* Correspondence: jorge.mejiamz@udlap.mx (J.L.M.-M.); hbach@mail.ubc.ca (H.B.); eugenio.sanchez@udlap.mx (E.S.-A.).

Figure S1. Chromatogram of hexane extract from *T. vanhouttei*.

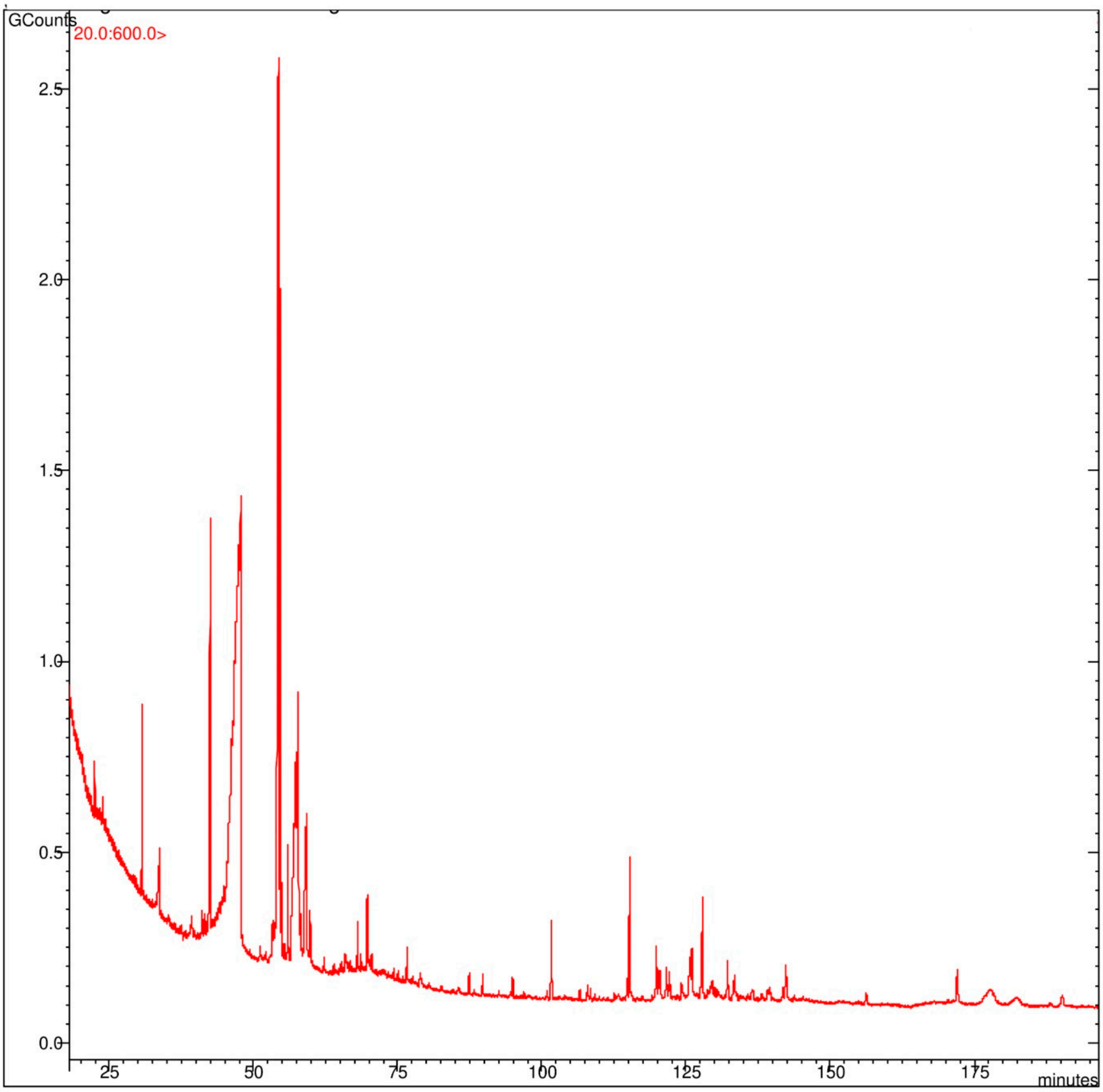

Figure S2. Chromatogram of chloroform extract from *T. vanhouttei*.

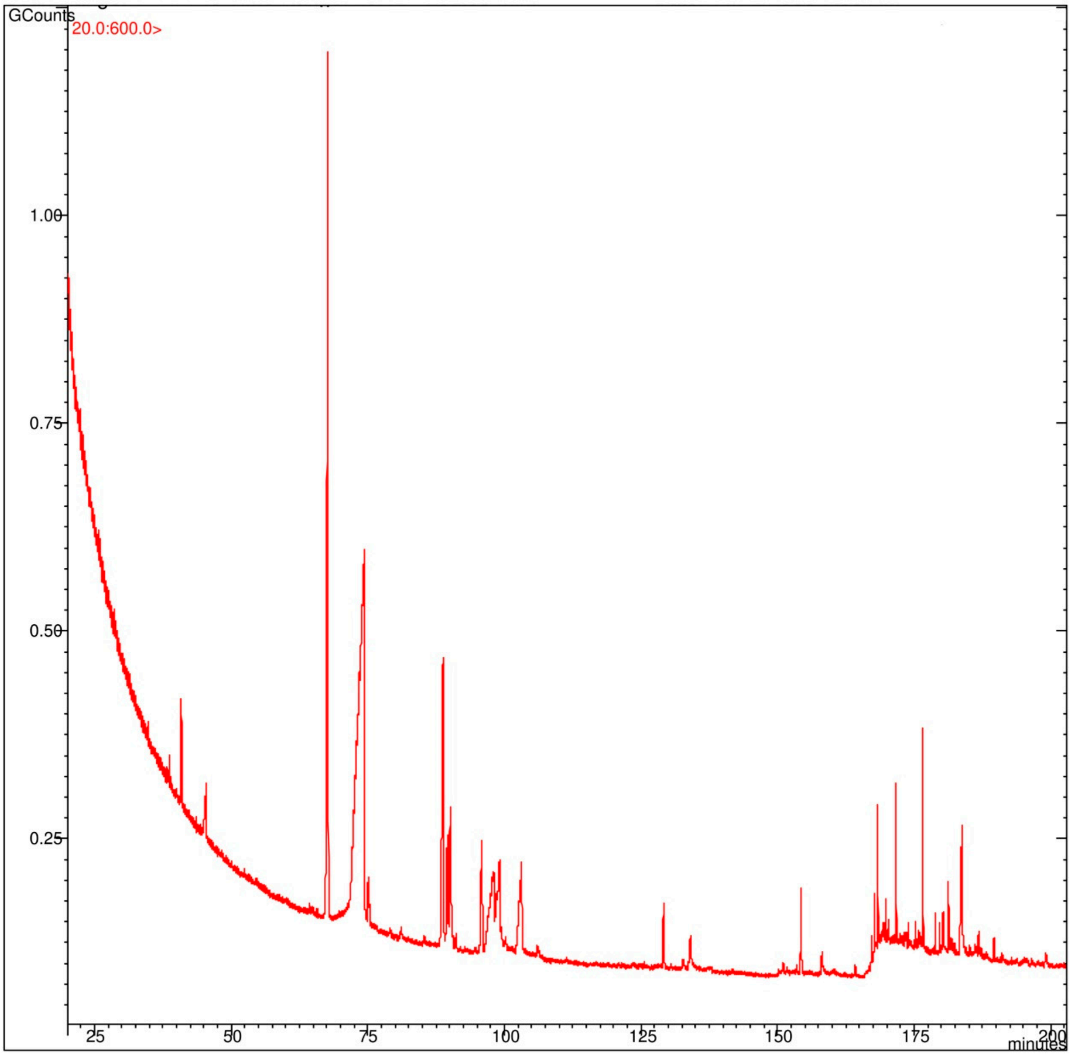

Figure S3. Chromatogram of methanol extract from *T. vanhouttei*.

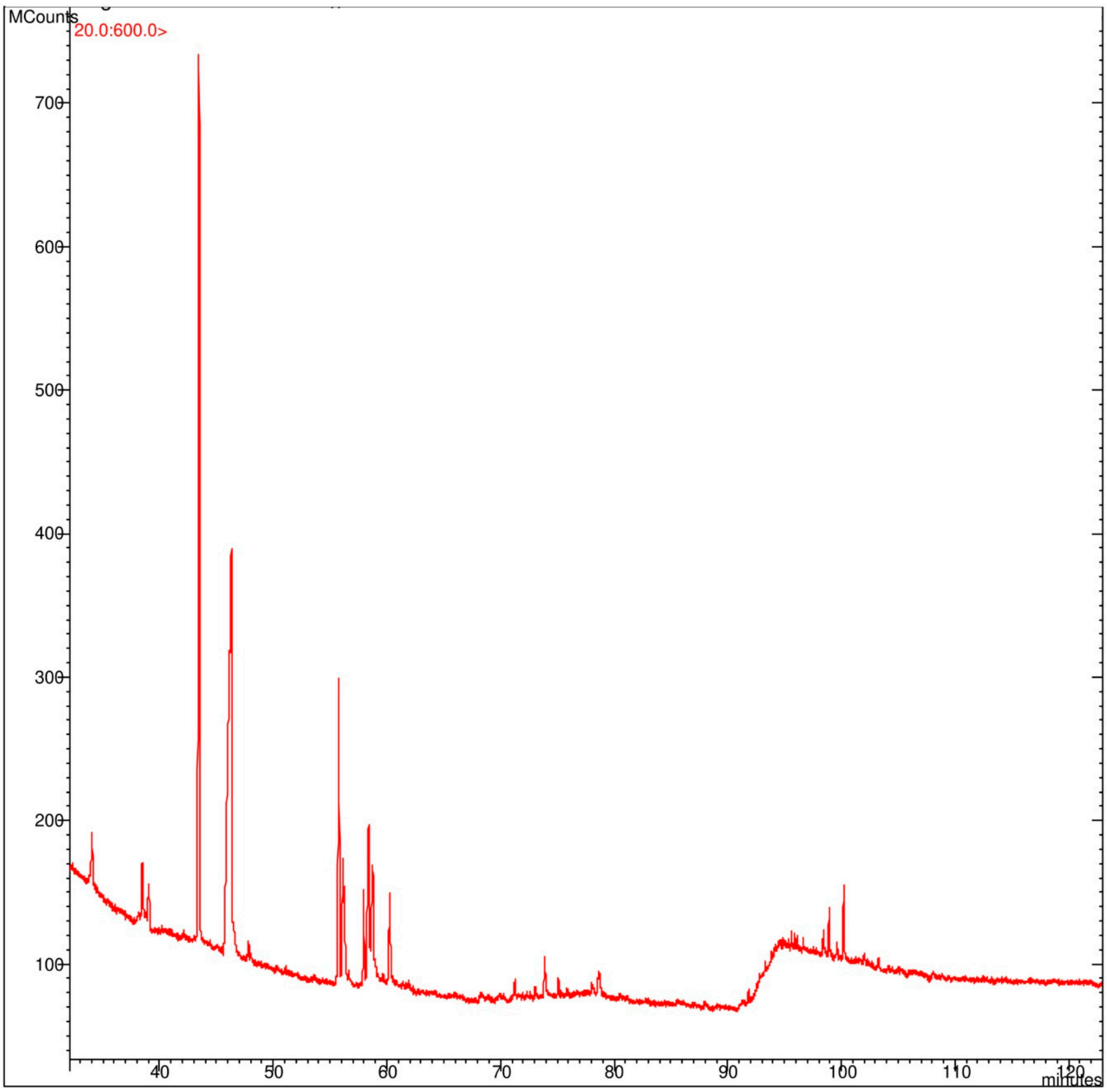

Supplement: Supplementary file 1 [file plants-12-03136-s001.zip › plants-2596622-supplementary.pdf]
